# Eicosapentaenoic Acid Improves Hepatic Metabolism and Reduces Inflammation Independent of Obesity in High-Fat-Fed Mice and in HepG2 Cells

**DOI:** 10.3390/nu11030599

**Published:** 2019-03-12

**Authors:** Kembra Albracht-Schulte, Samantha Gonzalez, Abigail Jackson, Savanna Wilson, Latha Ramalingam, Nishan S. Kalupahana, Naima Moustaid-Moussa

**Affiliations:** 1 Department of Nutritional Sciences and Obesity Research Cluster, Texas Tech University, Lubbock, TX 79409, USA; kembra.albracht@ttu.edu (K.A.-S.); samantha.m.gonzalez@ttuhsc.edu (S.G.); abigail.jackson@ttu.edu (A.J.); savanna.wilson@ttu.edu (S.W.); latha.ramalingam@ttu.edu (L.R.); skalupahana@pdn.ac.lk (N.S.K.); 2 Department of Physiology, University of Peradeniya, 20400 Peradeniya, Sri Lanka

**Keywords:** eicosapentaenoic acid, inflammation, nonalcoholic fatty liver disease, obesity, omega-3 polyunsaturated fatty acids

## Abstract

The prevalence of nonalcoholic fatty liver disease (NAFLD) is increasing worldwide, concurrent with increased obesity. Thus, there is urgent need for research that can lead to effective NAFLD prevention/treatment strategies. Omega-3 polyunsaturated fatty acids (n-3 PUFAs), including eicosapentaenoic acid (EPA), improve inflammation- and dyslipidemia-related metabolic disorders; however, mechanisms mediating the benefits of n-3 PUFAs in NAFLD treatment are less understood. We previously reported that EPA reversed obesity-induced hepatic steatosis in high-fat (HF)-fed B6 mice. Utilizing a combination of biochemical analyses of liver tissues from HF and HF-EPA-fed mice and a series of in vitro studies in tumor necrosis factor-alpha (TNF-α)-stimulated HepG2 cells, we dissect the mechanistic effects of EPA in reducing hepatic steatosis, including the role of EPA-targeted microRNAs (miRNA). With EPA, hepatic lipid metabolism was improved in HF-EPA mice, as indicated by decreased protein and messenger RNA (mRNA) levels of fatty acid synthase (FASN) and acetyl-CoA carboxylase (*Acaca*) gene, and increased mRNA levels for the peroxisome proliferator activated receptor-α (*Pparα*), and carnitine palmitoyltransferase (*Cpt*) 1a and 2 genes in the HF-EPA mice. Additionally, inflammation was reduced, as shown by decreased tumor necrosis factor-alpha (*Tnfα*) gene expression. Accordingly, EPA also significantly reduced *FASN* and *ACACA* mRNAs in human HepG2 cells. Glycolysis, estimated by extracellular acidification rate, was significantly reduced in HepG2 cells treated with EPA vs. vehicle. Furthermore, we identified several miRNAs that are regulated by EPA in mouse liver, including miR-19b-3p, miR-21a-5p, and others, which target lipid metabolism and inflammatory pathways. In conclusion, our findings provide novel mechanistic evidence for beneficial effects of EPA in NAFLD, through the identification of specific genes and miRNAs, which may be further exploited as future NAFLD therapies.

## 1. Introduction

Nonalcoholic fatty liver disease (NAFLD) is considered as the hepatic manifestation of the metabolic syndrome, due its bi-directional relationship with obesity, dyslipidemia, hypertension, and type 2 diabetes mellitus (T2DM) [1]. Prevalence of NAFLD and other metabolic consequences of obesity are increasing as obesity rates continue to increase [2]. 

Hyperinsulinemia and elevated blood glucose increase the release of free fatty acids (FFAs) and pro-inflammatory cytokines from enlarged adipocytes in the case of excessive energy intake, which negatively impacts surrounding organs, particularly the liver [3,4,5]. In addition to insulin resistance-driven increases in hepatic de novo lipogenesis, FFAs fluxed from adipose tissue may contribute to hepatic triglyceride (TAG) accumulation resulting in simple steatosis, which is the first stage in the development of NAFLD [6,7,8]. Lipotoxicity causes hepatocyte damage and leads to nonalcoholic steatohepatitis (NASH) development, via the promotion of inflammation and collagen deposition [9,10]. Further, disruptions in endocrine function related to increased adiposity and insulin resistance contribute to NASH development and progression in addition to other mechanisms, including oxidative stress [11,12]. 

Currently, lifestyle modification is the primary treatment for NAFLD [13]. No drug has been clinically approved; however, pharmaceuticals as well as dietary bioactive compounds that reduce inflammation and alter hepatic metabolism are under investigation for treatment of NAFLD [14]. Fish oil, rich in omega-3 (n-3) polyunsaturated fatty acids (PUFAs) such as eicosapentaenoic acid (C20:5n-3, EPA), is anti-inflammatory primarily by reducing production of pro-inflammatory cytokines [15]. Additionally, n-3 PUFAs are also effective at lowering triglycerides (TAGs) by targeting genes controlling lipogenesis [15]. Our lab has previously demonstrated that EPA supplementation (36 g/kg) along with high-fat (HF) diet improved metabolic parameters and significantly decreased hepatic TAG accumulation, when HF-fed mice (6-weeks) were switched to a HF diet enriched with EPA midway through the 12-week study (HF-EPA; 5-weeks). Interestingly, the HF and HF-EPA groups were similar in body weight, suggesting that EPA could improve the adverse effects of a HF diet independent of obesity [16]. 

Recent studies have suggested that reductions in inflammation and alterations in metabolism may be mediated by n-3 PUFAs targeting microRNAs (miRNA) related to these pathways [17,18,19]. miRNAs are non-coding RNAs that post-transcriptionally regulate gene expression by acting as sequence-specific inhibitors of messenger RNA (mRNA) [20]. Few miRNAs have been identified as key regulators in NAFLD pathogenesis [21,22,23,24]. However, no study has examined the influence of n-3 PUFAs on the expression of these miRNAs related to NAFLD. 

Thus, the purpose of this research was to understand obesity-independent mechanisms mediating the effects of EPA in the improvement of hepatic inflammation and lipid accumulation. We hypothesized that EPA directly/indirectly impacts steatosis by altering genes and miRNA related to lipid and carbohydrate metabolism as well as inflammatory processes and tested these effects in liver tissue of mice from the HF and HF-EPA groups as well as in HepG2 human hepatoma cells.

## 2. Materials and Methods

### 2.1. Animal Studies

The experimental groups and design used in this study have been previously described [16]. Briefly, male C57BL/6J mice aged 5–6 weeks were fed a HF diet (45% kcal from fat) for 11 weeks or the HF diet for the first 6 weeks and then a HF diet supplemented with 36 g/kg EPA ethyl ester for the remaining 5 weeks (HF-EPA) to examine the effectiveness of EPA in reversal of HF diet-induced obesity. These two groups of mice had comparable body weights and adiposity [16]. Given that the primary focus of this research was to dissect beneficial hepatic mechanisms of EPA related to obesity and inflammation, the low-fat (LF) group [16] was not included in the current study. Male mice were utilized since they are more prone to diet-induced metabolic complications compared to female mice [25]. Detailed diet information is provided in Supplementary Table S1 [16,26]. Mice were housed at 22 °C and food intake and body weight were measured daily. At the end of 11 weeks, mice were feed deprived for 4 h then euthanized using the CO_2_ inhalation method. Livers of these mice were collected as previously described [16] and were used for subsequent analyses. The Institutional Animal Care and Use Committee of the University of Tennessee, Knoxville, TN, where these studies were conducted, approved all the procedures (IACUC # 678). 

### 2.2. Liver Fatty Acid Composition, Liver Histology, and Liver Triglycerides

Direct fatty acid methyl ester (FAME) synthesis and the gas chromatography/mass spectrometry method previously described [27,28] were utilized to identify liver fatty acid concentrations and to validate EPA delivery to the liver. Frozen sections from the harvested livers were routinely fixed and stained with Oil Red-O (Sigma-Aldrich, St. Louis, MO, USA) and counterstained with Mayer’s Hematoxylin (ScyTek Laboratories Inc., Logan, Utah, USA). To perform the staining, the slides were immersed in formalin for 10 min, dipped in 60% isopropanol, covered in Oil Red-O for 15 min, dipped in 60% isopropanol, rinsed in water, counterstained with hematoxylin for 3 min, immersed briefly in Bluing Reagent (ScyTek Laboratories Inc., Logan, UT, USA), rinsed with water, and mounted. Lipids were imaged at 20× magnification using a cell-imaging microscope. Liver TAGs were measured as described previously (L-Type TAG M kit, Wako Chemicals USA, Inc., North Chesterfield, VA, USA) and normalized to liver weight [16,29]. 

### 2.3. Cell Culture

Human hepatoma HepG2 cells (ATCC, Manassas, VA, USA) were grown in Dulbecco’s modified Eagle’s medium (DMEM; Thermo Fisher, Pittsburgh, PA, USA) containing 10% fetal bovine serum (FBS; Atlanta Biologicals, Norcross, GA, USA) and 1% Penicillin–Streptomycin–Neomycin Antibiotic Mixture (Thermo Fisher, Pittsburgh, PA, USA) at 37 °C in 5% CO_2_. HepG2 cells were treated with various concentrations (25–100 μM) of EPA (Nu-chek Prep, Inc., Elysian, MN, USA) conjugated with fatty acid free bovine serum albumin (BSA; Sigma-Aldrich, St. Louis, MO, USA) or BSA for 24 h and 48 h. Based on pilot studies, we used 50 μM for 24 h and it did not affect cell viability (not shown) but was effective on metabolic responses studied and thus used in additional experiments. In order to replicate inflammation induced by HF feeding in the mouse study, HepG2 cells were treated with 25 ng/mL tumor necrosis factor-alpha (TNF-α) (Sigma-Aldrich, St. Louis, MO, USA) for 4 h prior to being treated with TNF-α supplemented with 50 μM EPA for 6 h. 

### 2.4. Gene Expression

Total RNA from liver as well as HepG2 cells was extracted using the Qiagen RNeasy kit (Qiagen, Valencia, CA, USA) and cDNA synthesis was performed using the iScript kit (Bio-Rad, Hercules, CA, USA). Gene expression was assessed by quantitative reverse transcription (RT)-PCR analysis (Bio-Rad, Hercules, CA, USA) using the Sybr Green PCR Master Mix (Bio-Rad, Hercules, CA, USA). The primers used in the gene expression analyses were purchased from Sigma-Aldrich, St. Louis, MO, USA. *Gapdh*/*18s* were used as the housekeeping genes for animal studies and *18s* was used as the housekeeping gene for cell treatment gene expression studies.

### 2.5. Immunoblotting

Proteins were extracted from livers by lysing in modified radio-immunoprecipitation (RIPA) assay buffer (Thermo Fisher, Pittsburgh, PA, USA). Protein was loaded in equal amounts per lane and separated using Mini-PROTEAN^®^ TGX Stain-Free™ Gels (Bio-Rad, CA, USA) and transferred to a polyvinylidene fluoride (PVDF) membrane using Immobilon-FL Transfer Membranes (MilliporeSigma, Burlington, MA, USA). The PVDF membrane was blocked using Pierce™ Protein-Free Blocking Buffer (Thermo Fisher, Pittsburgh, PA, USA) for an hour followed by incubation with primary antibodies for fatty acid synthase (FASN) (Santa Cruz Biotechnology, CA, USA; dilution 1:1000) and phosphorylated (Thr172) [30] and total AMP-activated protein kinase (AMPK) (Cell Signaling Technologies, MA, USA; dilution 1:500). Protein concentrations were normalized to beta-actin (β-actin) (Santa Cruz Biotechnology, CA, USA; 1:500). Mouse polyclonal antibody was used as a secondary antibody for FASN and β-actin (dilution 1:25,000), and rabbit polyclonal antibody was used as a secondary antibody for AMPK (dilution 1:25,000).

### 2.6. Respiration Measurements

Glycolytic rate for basal conditions and compensatory glycolysis following mitochondrial inhibition were measured utilizing the Seahorse XF^e^24 Flux Analyzer (Glycolytic Rate Assay Kit, Seahorse Bioscience, Agilent Technologies, MA, USA). HepG2 cells were seeded at a density of 40,000 cells per well onto 24-well XF cell culture microplates (Seahorse Bioscience) that had been coated with 50 μg/mL collagen diluted in 0.02 M acetic acid. The cells were allowed to grow to confluence and then were pretreated with either 50 µM EPA (Nu-chek Prep, Inc., Elysian, MN, USA) that had been conjugated with 1% bovine serum albumin (BSA, Sigma-Aldrich, St. Louis, MO, USA) or with 1% BSA alone and the 1% Penicillin–Streptomycin–Neomycin Antibiotic Mixture (Thermo Fisher, Pittsburgh, PA, USA) for 24 h. Assays were run according to instructions provided by the manufacturer. 

### 2.7. miRNA Analyses

Preliminary global miRNA profiling on liver tissue from the HF and HF-EPA groups was performed in collaboration with the University of Houston. Methods are described elsewhere [31]. Candidate miRNAs from this profiling were further validated through real time PCR analyses. Briefly, TaqMan^®^ Advanced miRNA cDNA Synthesis Kit (Thermo Fisher, Pittsburgh, PA, USA) was used for miRNA cDNA synthesis using samples from liver tissue from the HF and HF-EPA groups as well as HepG2 cells collected from the described treatment. miRNA expression was detected in the HF and HF-EPA groups as well as the treated HepG2 cells utilizing RT-PCR (Bio-Rad, Hercules, CA, USA), TaqMan^®^ Fast Advanced Master Mix (Thermo Fisher, Pittsburgh, PA, USA) and TaqMan^®^ Advanced miRNA Assays, (Thermo Fisher, Pittsburgh, PA, USA). miR-191-5p was used as the housekeeping miRNA for both in vivo and in vitro studies. 

### 2.8. Statistical Analyses

Results are presented as means ± standard error of the mean (SEM). Data were analyzed by performing *t*-tests when comparing two groups. Differences are considered significant at *p* < 0.05. Three to five replicates were used from the HF and HF-EPA groups. Cell culture experiments were repeated at least three times with triplicates within each experiment.

## 3. Results

As previously reported, the HF and HF-EPA groups were similar in body weight at the conclusion of the 11-week study. Mouse characteristics are presented in Supplementary Table S2. There were no significant differences in food intake between the two groups [16]. Fatty acid analysis of liver tissue from the HF and HF-EPA groups revealed enrichment with dietary EPA (Table 1). Liver tissue EPA enrichment paralleled previously reported red blood cell enrichment, while other fatty acids were not significantly different between the two groups [16]. 

### 3.1. EPA Reduces Hepatic Steatosis

**Figure 1:**
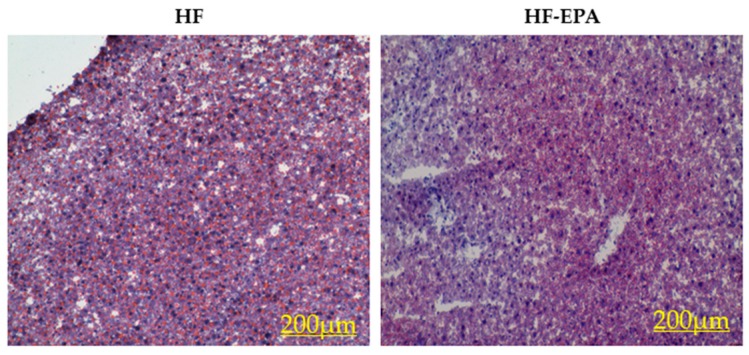
Effects of eicosapentaenoic acid (EPA) supplementation in the high-fat (HF) diet on liver histology. Representative Oil Red O staining of liver sections from mice fed the HF and HF-EPA diets, *n* = 3.

Oil Red O staining was used to assess TAG accumulation in the HF and HF-EPA groups (Figure 1). The HF-EPA group showed significantly reduced hepatic lipid accumulation as well as TAG levels (previously reported) [16]. 

### 3.2. EPA Regulates Hepatic Lipid Metabolism

**Figure 2:**
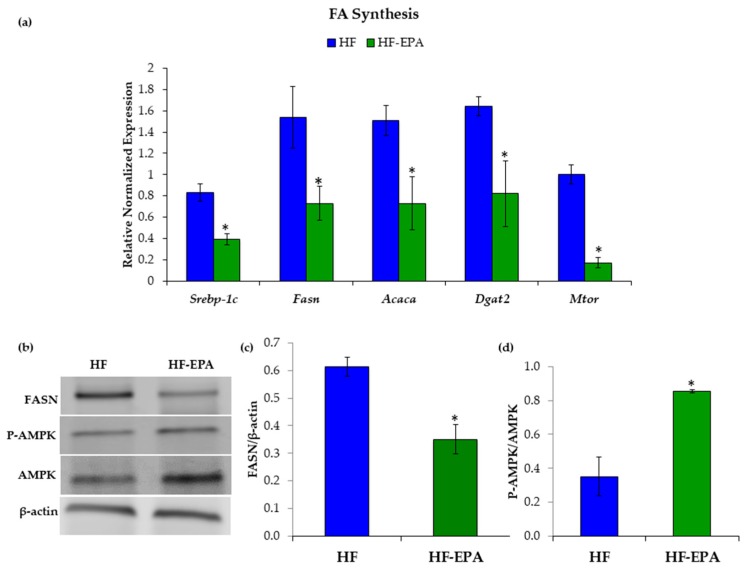
Eicosapentaenoic acid (EPA) supplementation reduces hepatic fatty acid (FA) synthesis. (**a**) Gene expression studies indicate significantly decreased markers of FA synthesis: sterol regulatory element-binding protein-1c (*Srebp-1c*), fatty acid synthase (*Fasn*), acetyl-CoA carboxylase (*Acaca*), diglyceride acetyltransferase (*Dgat2*), and mechanistic target of rapamycin (*Mtor*) with EPA supplementation. (**b**) Representative western blot images. Immunoblotting indicated decreased fatty acid synthase (FASN) protein levels and increased phosphorylated (Thr 172) AMP-activated protein kinase (P-AMPK) protein levels in livers of high-fat (HF)-EPA mice compared to mice fed the HF diet. β-actin served as the control. (**c**) Protein quantification indicated reduced FASN protein in HF-EPA group. (**d**) Protein quantification indicated increased P-AMPK protein in HF-EPA group, relative to total-AMPK. Data are expressed as mean ± SEM, *n* = 6, * = *p* < 0.05.

As EPA reduced hepatic TAG accumulation, we next wanted to determine the effects of EPA on hepatic lipid metabolism. To determine whether EPA beneficially regulates hepatic lipid metabolism, we used livers from mice fed a HF diet with or without EPA (HF-EPA). Gene expression levels of known contributors to TAG synthesis, including sterol regulatory element-binding protein-1c (*Srebp-1c*), fatty acid synthase (*Fasn*), acetyl-CoA carboxylase (*Acaca*), diacylglycerol O-acyltransferase 2 (*Dgat2*), and mechanistic target of rapamycin (*Mtor*) were significantly reduced in the HF-EPA group (Figure 2a). Additionally, we demonstrated that FASN protein content was significantly decreased in (HF-EPA) group compared to HF-fed mice. (Figure 2b,c). Furthermore, western blotting demonstrated significant increases in AMP-activated protein kinase (AMPK) protein content (Figure 2b,d), which negatively regulates Srebp-1c, a known up-regulator of TAG synthesis [32]. Phosphorylated-AMPK was normalized to total-AMPK.

**Figure 3:**
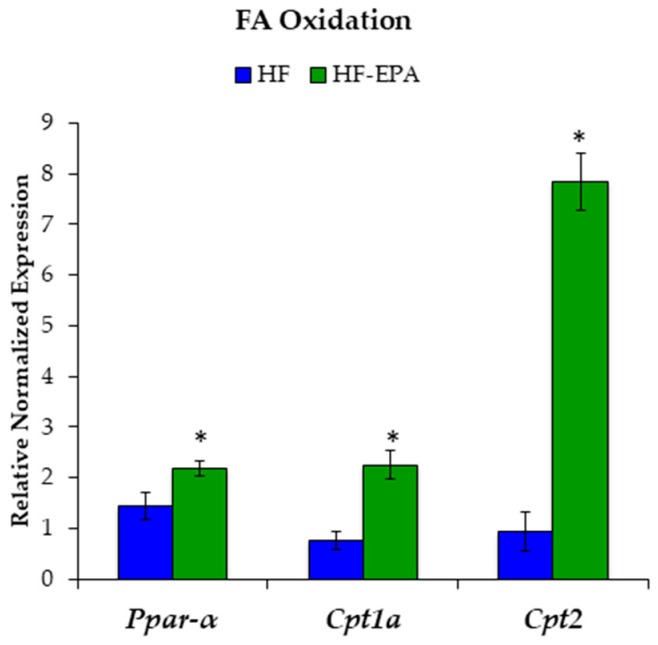
Eicosapentaenoic acid (EPA) supplementation increases hepatic fatty acid (FA) oxidation. Gene expression studies indicate significant increases in markers of FA oxidation: peroxisome proliferator-activated receptor-alpha (*Pparα*), carnitine palmitoyltransferase (*Cpt*)1a, and *Cpt2* with EPA supplementation. Data are expressed as mean ± SEM, *n* = 6, * = *p* < 0.05.

Since markers of lipogenesis were decreased, we then examined markers of fatty acid beta-oxidation (β-oxidation) to determine if EPA was causing a beneficial shift in hepatic lipid metabolism towards catabolism. Gene expression levels of fatty acid β-oxidation, including peroxisome proliferator-activated receptor-alpha (*Pparα*) and carnitine palmitoyltransferase (*Cpt*)1a and 2 were significantly increased with EPA (Figure 3). 

**Figure 4:**
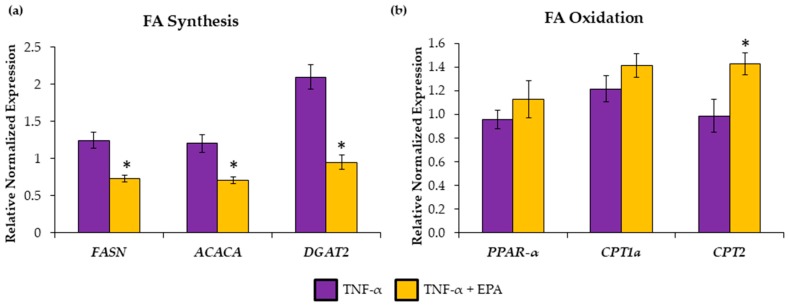
Eicosapentaenoic acid (EPA) supplemented to tumor necrosis factor-alpha (TNF-α)-treated HepG2 cells alters fatty acid (FA) metabolism. Gene expression studies indicate significant (**a**) decreases in markers of FA synthesis: fatty acid synthase (*FASN*), acetyl-CoA carboxylase (*ACACA*), and diglyceride acetyltransferase (*DGAT2*), and (**b**) increases in carnitine palmitoyltransferase (*CPT*)-2, a marker of fatty acid oxidation. Peroxisome proliferator-activated receptor-alpha (PPARα) and CPT1a were not significantly different between groups. Data are expressed as mean ± SEM, *n* = 6, * = *p* < 0.05.

To validate in vivo findings, we used HepG2 cells treated with 25 ng/mL TNF-α (to mimic HF diet-induced inflammation) and 50 μM EPA. Gene expression levels of *FASN*, *ACACA*, and *DGAT2* were significantly reduced when EPA was added to TNF-α (Figure 4a) compared to TNF-α treatment alone. However, *CPT2* was the only fatty acid β-oxidation mRNA tested that was significantly upregulated by EPA (Figure 4b).

### 3.3. EPA Regulates Hepatic Carbohydrate Metabolism

**Figure 5:**
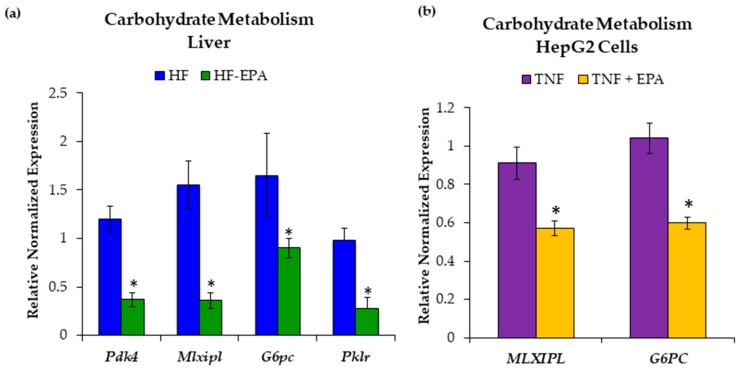
Eicosapentaenoic acid (EPA) supplementation alters carbohydrate metabolism. (**a**) Gene expression studies indicate significant decreases in gene expression of pyruvate dehydrogenase kinase (*Pdk4*), MLX interacting protein-like (*Mlxipl*), glucose-6-phosphatase (*G6pc*), and pyruvate kinase L/R (*Pklr*) in liver tissue of high-fat (HF)-EPA fed mice. Data are expressed as mean ± SEM, *n* = 6, *p* ≤ 0.05. (**b**) EPA supplemented to TNF-α treated HepG2 cells reduces carbohydrate metabolism. Gene expression studies indicate significantly decreases in markers of carbohydrate metabolism: MLX interacting protein-like (*MLXIPL*), and glucose-6-phosphatase (*G6PC*). Data are expressed as mean ± SEM, *n* = 6, * = *p* < 0.05.

Since elevated glucose and increased glycolysis can contribute to the production of acetyl-CoA, a key component in de novo lipogenesis [33], we next examined markers associated with hepatic carbohydrate metabolism. To determine whether EPA regulates carbohydrate metabolism in the liver, we used livers from the HF and HF-EPA mice. Gene expression levels for pyruvate dehydrogenase kinase 4 (*Pdk4*), MLX-interacting protein-like (*Mlxipl*), glucose-6-phosphatase catalytic subunit (*G6pc*), and pyruvate kinase L/R (*Pklr*), were significantly reduced with EPA (Figure 5a). 

To validate in vivo findings, we used HepG2 cells treated with TNF-α and EPA. Gene expression levels of *MLXIPL* and *G6PC* were significantly reduced when EPA was added to TNF-α (Figure 5b).

**Figure 6:**
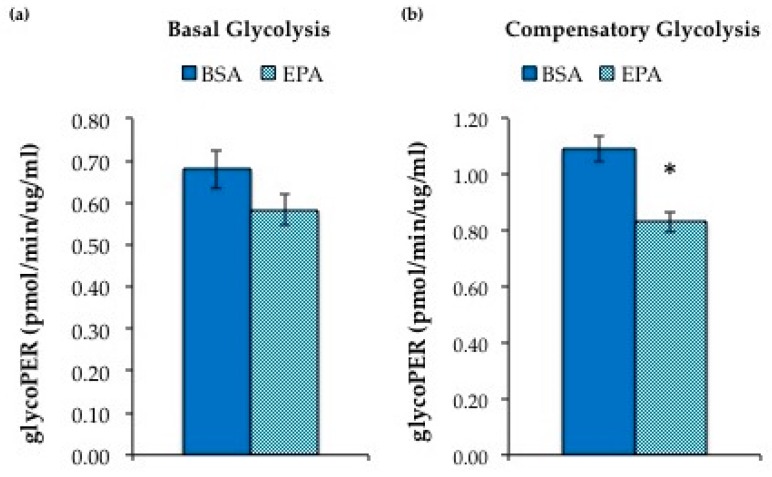
HepG2 cells treated with 50 µM eicosapentaenoic acid (EPA) showed a lower glycolytic phenotype compared to cells treated with bovine serum albumin (BSA). (**a**) Basal glycolysis was not significantly different between the two groups. (**b**) After inhibition of oxidative phosphorylation, EPA resulted in decreased compensatory glycolysis. Data are expressed as mean ± SEM, *n* = 4, * = *p* < 0.05.

Utilizing the Seahorse XF Glycolytic Rate Assay kit, we assessed glycolysis in HepG2 cells treated with 50μM EPA versus BSA. We found significantly reduced compensatory glycolysis (Figure 6), which is measured when oxidative phosphorylation is inhibited, indicating reduced glycolytic capacity in HepG2 cells treated with EPA. 

### 3.4. EPA Reduces Hepatic Inflammation

**Figure 7:**
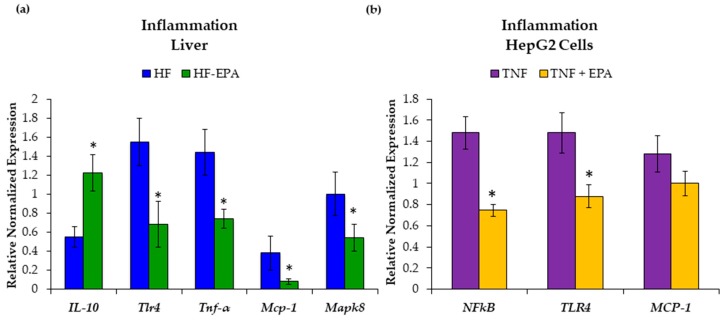
Eicosapentaenoic acid (EPA) supplementation reduces hepatic inflammation. (**a**) Gene expression studies indicate significant increases in anti-inflammatory marker, interleukin 10 (*Il-10*) and significant decreases in pro-inflammatory markers: toll-like receptor 4 (*Tlr4*), tumor necrosis factor-alpha (*Tnf-α*), monocyte chemoattractant protein-1 (*Mcp-1*), and mitogen activated protein kinase 8 (*Mapk8*) with EPA supplementation. Data are expressed as mean ± SEM, *n* = 6, *p* ≤ 0.05. (**b**) EPA supplemented to TNF-α treated HepG2 cells reduces inflammation. Gene expression studies indicate significant decreases in pro-inflammatory markers: nuclear factor kappa-light-chain-enhancer of activated B cells (*NFκB*) and toll-like receptor 4 (*TLR4*) with EPA supplementation. Reductions in monocyte chemoattractant protein-1 (*MCP-1*) were not significant. Data are expressed as mean ± SEM, *n* = 6, * = *p* < 0.05.

Mice fed the HF and HF-EPA diets were similar in body weight; thus, we next assessed markers of obesity-associated inflammation in the liver [3,16]. To determine whether EPA reduced inflammation directly in the liver, we used liver from mice fed a HF diet with or without EPA (HF-EPA). Gene expression analyses showed increased interleukin-10 (*Il-10*) expression, which is anti-inflammatory, in the HF-EPA group. Furthermore, gene expression analysis showed significantly decreased mRNA expression of monocyte chemoattractant protein 1 (*Mcp-1*), toll-like receptor 4 (*Tlr4*), TNF-α and mitogen activated protein kinase 8 (*Mapk8*) in the HF-EPA group (Figure 7a). 

To validate in vivo findings, we used HepG2 cells treated with TNF-α and EPA. Gene expression levels of *NF-κB* and *TLR-4* were decreased with the addition of EPA (Figure 7b). Decreases in *MCP-1* were not significant and expression of *MAPK8* was too low for analysis. 

### 3.5. EPA Regulates Hepatic miRNA Involved in Lipid Metabolism and Inflammation

MiRNA regulate various genes related to metabolism and inflammation [34], with few studies indicating n-3 PUFA to target miRNAs [35,36]. Therefore, we sought to determine differences in miRNA regulation by EPA in the livers of mice fed a HF diet. Global miRNA profiling in liver tissues of mice fed with or without EPA revealed about 30 miRNAs that were significantly different between the HF and HF-EPA samples (data not shown). Targets were selected based on >1.5-fold expression, statistical significance of *p* ≤ 0.05, and false discovery rate (FDR) ≤5%. miR-19b-3p, -21a-5p, and -101b-3p were identified for validation as they were documented to have a role in NAFLD [37,38,39]. Furthermore, miR-let7a-5p was chosen for validation for its known role in inflammation [40,41] and miR-455-5p was chosen for its novelty related to liver function. 

**Figure 8:**
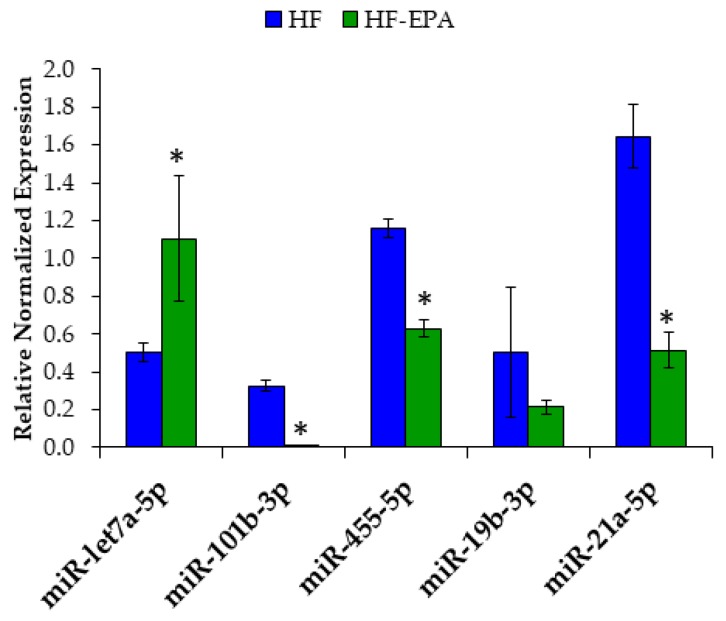
Eicosapentaenoic acid (EPA) supplementation significantly alters miRNA liver expression. miR-let7a-5p was significantly increased in the high-fat (HF)-EPA group; miR-101b-3p, -455-5p, and -21a-5p were significantly decreased with EPA; miR-19b-3p was significantly decreased in profiling but could not be validated. Data are expressed as mean ± SEM, *n* = 4–6, * = *p* < 0.05.

We found significantly increased expression of miR-let7a-5p in the HF-EPA group and significantly decreased expression of miR-21a-5p, miR-101b-3p and miR-455-5p (Figure 8) compared to HF. However, we were unable to validate significant decreases in miR-19b-3p. 

**Figure 9:**
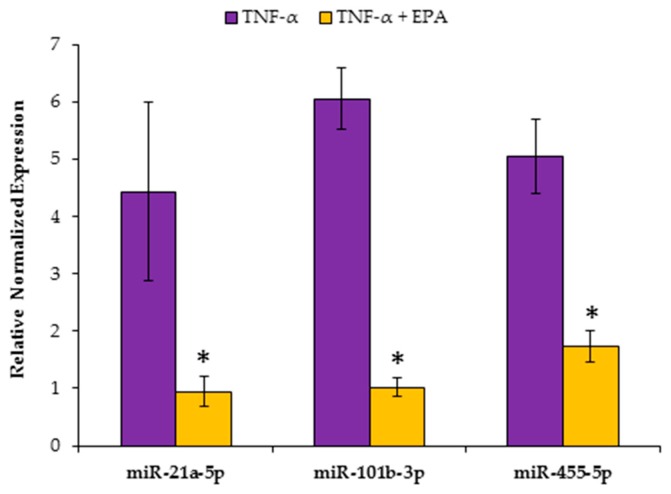
Eicosapentaenoic acid (EPA) alters miRNA expression in treated HepG2 cells. miR-21a-5p, -101b-3p, and -455-5p were significantly reduced with EPA in TNF-α stimulated HepG2 cells. Data are expressed as mean ± SEM, *n* = 4–6, * = *p* < 0.05.

To further validate the liver specific function of these miRNAs, we used HepG2 cells treated with TNF-α and EPA in order to mimic in vivo studies. Utilizing RT-qPCR, we found significant decreases in miR-21a-5p, miR-101b-3p and miR-455-5p (Figure 9). miR-let-7a-5p expression was too low for detection. 

## 4. Discussion

NAFLD is projected to be the leading cause of liver related morbidity and mortality within the next 20 years [42]. Therefore, therapeutic strategies are necessary to prevent metabolic derangements associated with obesity-related NAFLD since it is known to instigate and exacerbate insulin resistance and systemic inflammation [43].

In this study, we report (1) reduced hepatic TAG accumulation due to decreased fatty acid synthesis and upregulated β-oxidation, (2) reduced hepatic carbohydrate metabolism, and (3) reduced hepatic inflammation with EPA supplementation independent of body weight in C57BL6 mice fed a HF diet. Additionally, we were able to validate these findings using a HepG2 cell culture model of NAFLD and show that EPA has liver specific benefits in an inflammatory environment. To our knowledge, this is the first study to indicate that EPA targets hepatic miRNA involved in NAFLD pathways in order to improve metabolism and reduce inflammation. 

Excessive hepatic accumulation of TAG (≥5%) is the hallmark of NAFLD [7]. Increased dietary intake, hyperglycemia and hyperinsulinemia largely influence hepatic de novo lipogenesis [44]. Insulin upregulates liver X receptor (LXR), sterol regulatory element binding protein-1c (SREBP-1c) and carbohydrate response element binding protein (ChREBP), all of which are transcription factors that control glycolytic and lipogenic genes [5,45,46]. It is known that n-3 PUFAs alter expression and nuclear localization of these transcription factors and thus reduce TAG synthesis [47,48]. Accordingly, we have shown decreased *Srebp-1c* and ChREBP (*Mlxipl*) expression as well as decreases in lipogenic genes, including *Acaca* and *Fasn* and glycolytic genes, including pyruvate kinase L/R (*Pklr*) in the HF-EPA mice. Additionally, we also found decreases in *Dgat2* expression, the enzyme responsible for TAG synthesis [49]. Interestingly, Calo et al. demonstrated reduced expression of glycolytic and gluconeogenic genes in miR-21 knockout mice [50]. Therefore, our report of EPA-mediated decreases in miR-21a-5p in the HF-EPA group could be associated with decreases in the glycolytic and gluconeogenic genes seen in our study. In addition, metabolism studies from HepG2 cells treated with EPA indicated reductions in glycolysis.

Furthermore, we also report increased AMPK in the livers of mice fed a HF diet supplemented with EPA. AMPK is thought of as a “metabolic master switch” due to its concurrent inhibition of fatty acid synthesis, through phosphorylation of SREBP-1c [32] or raptor of the mTOR complex [51], and activation of catabolic pathways, including β-oxidation [52]. Indeed, activation of AMPK prevents steatosis [53,54]. Accordingly, we also found decreases in *Mtor* gene expression in the HF-EPA group. It has been suggested that mTOR activation is required for SREBP-1c activity related to lipogenesis, particularly related to insulin-mediated activity in the case of insulin resistance [55]. Our findings on hepatic lipogenesis and reduced TAG accumulation with EPA are particularly interesting since we initially reported reduced plasma insulin but unimproved glucose tolerance in the HF-EPA group [16]. Therefore, we suggest that EPA regulates lipogenesis by controlling genes involved in these pathways, independent of glycemic control but aided by reductions in insulin. 

Although there is debate on the role of miR-21 in relation to NAFLD [34,50,56], our findings indicate a beneficial decrease in miR-21a-5p with regard to NAFLD and agree with a recent study showing that miR-21 knockout mice had reduced hepatic steatosis and lipogenesis [50]. Indeed, serum levels of miR-21 are higher in individuals with NAFLD [38,57]. PPARα, a key transcriptional regulator of hepatic energy homeostasis, is a suggested target of miR-21, thus indicating an inverse relationship between the two [34,58]. It is well established that PUFAs can act as ligands for PPARs, including PPARα [59] and thus upregulate β-oxidation [60,61] and related mitochondrial enzymes [47]. In agreement, our study showed an increase in *Pparα* gene expression and related increases in mitochondrial enzymes *Cpt1a* and *Cpt2*, indicating a body weight-independent increase in hepatic β-oxidation in mice fed a HF diet supplemented with EPA. Moreover, we suggest that by targeting miR-21a-5p, EPA could have indirectly increased *Pparα* expression and increased hepatic fatty acid transport and β-oxidation. Others have also demonstrated that miR-21 is targeted by n-3 PUFA [35,36], but no other study has reported miR-21 to be targeted by EPA in relation to hepatic lipid metabolism. 

Inflammation can occur in the liver via (1) increased adipocyte release of pro-inflammatory cytokines that are delivered to the liver (i.e., as part of obesity-associated systemic inflammation) which may be worsened by insulin resistance; and/or (2) the influx of fatty acids from adipocytes overwhelms adaptive mechanisms causing hepatocyte dysfunction and injury in a process known as lipotoxicity [62]. Interestingly, we report reduced hepatic inflammation in the HF-EPA group despite similarities in body weight with mice fed HF diet. Reductions in *NF-κB* and pro-inflammatory cytokines, such as *Tnf-α* and *Mcp-1*, as well as increases in anti-inflammatory cytokines, such as *Il-10*, are unsurprising since n-3 PUFAs are well-established anti-inflammatory agents [63]; however, reporting improvements in inflammation independent of obesity is noteworthy. Furthermore, we were able to validate liver specific reductions in inflammation with EPA, via reductions in *NF-κB*, and *TLR4*.

Global miRNA profiling in livers from the HF and HF-EPA groups revealed significant increases in the miR-let-7 family, including miR-let7a-5p, -let7b-3p, -let7c-5p, and -let7d-5p (data not shown) with EPA. Of which, we selected and were able to validate significant increases in miR-let7a-5p with qPCR. While no study has indicated an n-3 mediated increase in the miR-let-7 family or a relationship with NAFLD, specifically, few studies have reported that miR-let-7 plays a role in the regulation of inflammatory pathways in cancer and atherosclerosis [40,41,64]. A feedback loop between miR-let-7 and transcriptional regulators such as NF-κB, as well as receptors including TNF-α and Tlr4, is consistently reported and indicate that an increases in NF-κB, for example, reduces miR-let-7, which results in the increased production of pro-inflammatory cytokines, such as IL-6 [40,41]. Therefore, increases in miR-let-7 would decrease production of pro-inflammatory cytokines. Furthermore, Xu et al. demonstrated a role for miR-let-7 in decreasing Ras activity in both NF-κB and MAPK pathways [64], thereby decreasing inflammation. In the MAPK/ERK/JNK pathway, JNK1 (also known as MAPK8) is known to promote the development of steatosis and inflammation [65]. Taken together with our gene expression studies, our findings indicate that EPA beneficially upregulates miR-let-7 and decreases the production of pro-inflammatory cytokines.

The one study that has examined the effect of n-3 PUFA intake on miRNA regulation reported n-3 PUFA mediated decreases in miR-19b-3p paired with decreases in markers of inflammation, including *Tlr4* and *Mapk* [18]. Our findings are in agreement with these findings; however, we cannot limit the activity of miR-19b-3p to the regulation of inflammation given other studies indicating negative regulation of PPARα and AMPK [37], as well as our findings that also suggest a role in increasing β-oxidation and reducing lipid synthesis. Collectively, this highlights the potential role of n-3 mediated miR-19b-3p regulation of inflammatory and lipid metabolism pathways related to NAFLD and should be investigated further. 

Lastly, we utilized a diet containing 36 g/kg EPA, which accounts for approximately 6.75% of total energy intake in the HF-fed mice [16]. This dosage exceeds the current recommendation of 1–4 g/day of n-3 PUFA in humans. However, human studies have been performed with doses up to 15g/day [15,66]. Thus, future studies are needed to examine the dose-dependent effects of EPA in obesity-related NAFLD. There are a few limitations to the current study. Only male mice were used in the current study, as they are more susceptible to diet-induced obesity and metabolic dysfunctions than females in this mouse strain. Thus, this limits translation to females [67] and warrants future studies. Another limitation to the current study is the lack of a low-fat (LF) group and comparison to LF-EPA-fed mice, which also requires future studies. 

## 5. Conclusions

**Figure 10:**
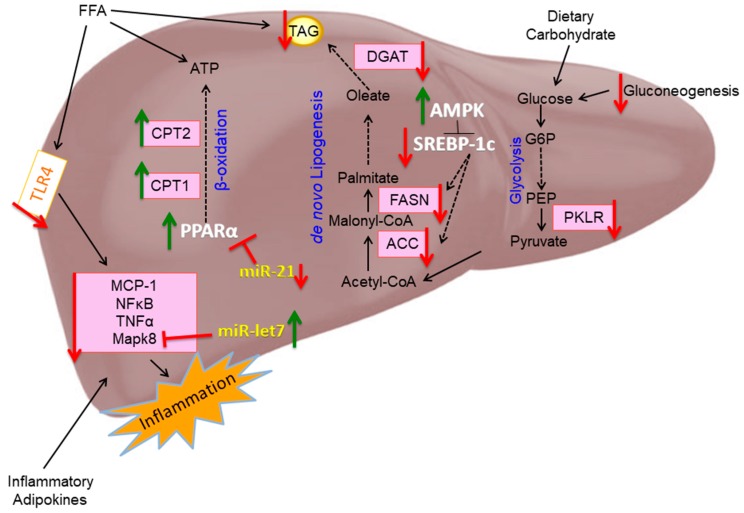
Proposed model of mechanistic activity of eicosapentaenoic acid (EPA) in reducing hepatic triglyceride (TAG) accumulation. EPA reduces glycolysis by reducing pyruvate kinase L/R (PKLR) and gluconeogenesis and thus the amount of glucose available for de novo lipogenesis. Further, TAG synthesis is reduced with EPA, through activation of AMP-activated protein kinase (AMPK), resulting inhibition of sterol regulatory element-binding protein-1c (SREBP-1c) and reduction of enzymes, fatty acid synthase (FASN), acetyl-CoA carboxylase (ACC), and diglyceride acetyltransferase (DGAT2). Utilization of free fatty acids (FFA) for adenosine triphosphate (ATP) generation is upregulated with EPA through induced expression of peroxisome proliferator-activated receptor-alpha (PPARα) and carnitine palmitoyltransferase (CPT)-1a and CPT2. Additionally, EPA reduces hepatic inflammation by reducing expression of toll-like receptor 4 (TLR4), as well as the production of pro-inflammatory cytokines, monocyte chemoattractant protein-1 (MCP-1), nuclear factor kappa-light-chain-enhancer of activated B cells (NFκB), tumor necrosis factor-alpha (TNF-α), and mitogen activated protein kinase 8 (Mapk8). MicroRNA, including miR-21 and miR-let7a-5p are targeted by EPA and may contribute to improved hepatic metabolism and reduced inflammation.

In conclusion, our findings demonstrate that EPA exhibits protective effects in liver steatosis and inflammation that may be mediated by genes and miRNAs in lipid and carbohydrate metabolism as well as inflammatory pathways (Figure 10). 

Given the side effects and limitations of pharmacological therapies, these findings provide the foundation for novel therapeutics related to n-3 PUFAs in improving metabolism and inflammation in the liver. However, future studies are necessary to understand the role of these n-3 PUFA-targeted miRNAs as novel therapies in the liver and other tissues in relation to metabolic diseases. 

## Figures and Tables

**Table 1:** Fatty acid composition of liver tissue in mice from HF and HF-EPA groups.

	HF	HF-EPA	*p*-Value
Palmitic acid 16:0	29.02 ± 3.50	25.73 ± 1.36	0.43
Palmitoleic acid 16:1 (n-7)	3.76 ± 0.60	2.91 ± 1.08	0.53
**PUFA**			
*n-6 PUFAs*			
Linoleic acid 18:2 (n-6)	19.93 ± 3.42	12.55 ± 5.19	0.30
Arachidonic acid 20:4 (n-6)	7.10 ± 1.31	5.30 ± 1.45	0.40
*n-3 PUFAs*			
Linolenic acid 18:3 (n-3)	0.65 ± 0.12	0.91 ± 0.17	0.29
Eicosapentaenoic acid 20:5 (n-3)	0.22 ± 0.12	8.15 ± 1.07	0.001 *
Docosahexaenoic acid 22:5 (n-3)	3.64 ± 0.86	5.46 ± 1.43	0.34

Results are mean ± SEM. * = *p* < 0.05. PUFA: polyunsaturated fatty acid.

## Supplementary Materials

The following are available online at https://www.mdpi.com/2072-6643/11/3/599/s1, Table S1: Diet Composition, Table S2. Characteristics in C57BL/6J HF and HF-EPA Mice.
