# Supplementary material for: Eicosapentaenoic Acid Improves Hepatic Metabolism and Reduces Inflammation Independent of Obesity in High-Fat-Fed Mice and in HepG2 Cells
Source: Nutrients. 2019 Mar 12;11(3):599. doi: 10.3390/nu11030599 (PMC6471632; doi:10.3390/nu11030599)
Supplement: Supplementary file 1 [file nutrients-11-00599-s001.pdf]

**Table 1.** Diet Composition [16].

| | HF | HF-EPA |
|---|---|---|
| **Fat, % energy** | 45 | 45 |
| **Carbohydrate, % energy** | 35 | 35 |
| **Protein, % energy** | 20 | 20 |
| **Digestible energy, kJ/g** | 19.8 | 19.8 |
| | **g/kg** | |
| **Lard** | 207 | 171 |
| **Soybean oil** | 29 | 29 |
| **EPA, ethyl ester** | 0 | 36 |
| **Mineral mix, S10026** [1] | 12 | 12 |
| **Dicalcium phosphate** | 15 | 15 |
| **Calcium carbonate** | 6 | 6 |
| **Potassium citrate, 1 $H_2O$** | 19 | 19 |
| **Vitamin mix, V10001** [1] | 12 | 12 |
| **Choline bitartrate** | 2 | 2 |
| **dl-$\alpha$-Tocopheryl acetate** | 0.15 | 0.15 |
| | **% total fatty acids** | |
| **SFA** | 36.3 | 30.0 |
| 14:0 | 0.8 | 0.7 |
| 16:0 | 22.6 | 18.7 |
| 18:0 | 12.9 | 10.6 |
| **Monounsaturated fats** | 45.3 | 37.6 |
| 14.1 | 0.5 | 0.4 |
| 16:1(n-9) | 3.5 | 2.9 |
| 18:1(n-9) | 41.3 | 34.3 |
| **PUFA** | 18.5 | 32.4 |
| 18:2(n-6) | 14.9 | 13.5 |
| 18:3(n-3) | 1.9 | 1.8 |
| 20:4(n-6) | 1.6 | 1.3 |
| 20:5(n-3) | 0.0 | 16.1 |

[1] Research Diets; composition of mineral and vitamin mix as described previously by DeFuria et al. [26]

**Table 2.** Characteristics in C57BL/6J HF and HF-EPA Mice.

| | HF | HF-EPA | *p*-value |
|---|---|---|---|
| Dead weight, g | 40.14 ± 1.2 | 40.6 ± 1.1 | NS |
| Adiposity Index, % | 14.8 ± 0.4 | 15.6 ± 0.5 | NS |
| Plasma TG, mmol/L | 0.48 ± 0.03 | 0.39 ± 0.03 | 0.04 |
| Liver TG [1], μmol/g | 82 ± 2.4 | 54 ± 6.2 | <0.001 |

Reproduced with permission from Oxford University Press (license # 4534910231611; [16]). Results are represented as mean ± SEM, $n$ = 9-10. [1] $n$ = 4
